# Influences of Distributive Injustice and Job Insecurity Amid COVID-19 on Unethical Pro-Organisational Behaviour: Mediating Role of Employee Turnover Intention

**DOI:** 10.3390/ijerph19127040

**Published:** 2022-06-08

**Authors:** Meqbel M. Aliedan, Abu Elnasr E. Sobaih, Mansour A. Alyahya, Ibrahim A. Elshaer

**Affiliations:** 1 Management Department, College of Business Administration, King Faisal University, Al-Ahsaa 31982, Saudi Arabia; maliedan@kfu.edu.sa (M.M.A.); malyahya@kfu.edu.sa (M.A.A.); 2 Hotel Management Department, Faculty of Tourism and Hotel Management, Helwan University, Cairo 12612, Egypt; 3 Hotel Studies Department, Faculty of Tourism and Hotel Management, Suez Canal University, Ismailia 41522, Egypt

**Keywords:** job insecurity, distributive injustice, COVID-19, unethical pro-organisational behaviour, turnover intention, hotel industry, Kingdom of Saudi Arabia

## Abstract

Drawn on Social Exchange Theory and Conservation of Resources Theory, this study developed a research model to examine the direct influence of job insecurity and distributive injustice, which were common in many hotels amid COVID-19, on unethical pro-organisation behaviour (UPoB) among hotel employees. The study also examines the mediating role of turnover intention in the relationship between job insecurity, and distributive injustice, which was result of the COVID-19 pandemic on UPoB. For this purpose, a questionnaire survey was self-dropped and collected through personal network to hotel employees in Eastern Province of Saudi Arabia. The results of structural equation modelling using AMOS (version 23) supported all the study hypotheses. The results showed a significant positive influence of distributive injustice and job insecurity on UPoB among hotel employees. Moreover, turnover intention was found to have a partial mediation role in the relationship between job insecurity, distributive injustice and UPoB. The results extend our understanding of Social Exchange Theory and Conservation of Resources Theory that employees in hotels are more likely to protect themselves and their job by engaging in UPoB if they perceived their job at threat due to a crisis, i.e., the COVID-19 pandemic. The major conclusion of current research is that when hotels employees perceived job insecurity and distributive injustice because of the pandemic, they responded with high turnover intention and as a last choice engaging in UPoB to save their resources, in this case their jobs, since they have no other alternatives outside the organisations. However, this inappropriate antisocial behaviour could have a negative influence on both employees and organisation at the long term. The results of current research have several theoretical implications for tourism scholars and managerial implication for hoteliers.

## 1. Introduction

The Corona Virus Disease (COVID-19) pandemic has severely hit the international economy, including the tourism industry, since the first quarter of 2020. Hotels have been among the hard-hit sectors [1]. Hotel employees were affected by the COVID-19 pandemic due to business disturbance and mass layoffs [2]. The pandemic has had several physiological and psychological impacts on frontline employees, who are in direct contact with people, e.g., most of the hotel employees. Due to the pandemic, studies [1,2,3,4,5] showed that hotel employees have felt stressed, less secure, and worried about their job. Job insecurity, distributive injustice, and turnover intention were some examples of common perceptions among hotel employees due to the COVID-19 pandemic [4], which often lead to the practice of unethical behaviour [6]. However, a recent study by Alyahaya et al. [4], noted limited research studies but growing, on the physiological and psychological impact of COVID-19 on hotel employees. This study is an attempt to bridge this gap in knowledge in relation to the influences of job insecurity, distributive injustice, and turnover intention on unethical pro-organizational behaviour (UPoB).

The antecedences and consequences of unethical behaviour have been studied comprehensively over the last few decades [6,7,8,9]. The previous studies often refer to unethical behaviour as any illegal and/or immoral actions or practices, which are performed by employees for their own self-interest [10]. Nonetheless, other research [10] considered unethical behaviour as any practice that is considered improper for an individual, team, or organisation. This includes benefiting others in the workplace to receive benefits in return [10]. It also includes dishonest and lying behaviour to benefit others gain from this action [11]. This unethical behaviour can also be undertaken in the name of the team or the organization to which an employee belongs [7]. This is what is so-called unethical pro-organizational behaviour [10]. 

Unethical pro-organizational behaviour is to undertake any unethical action or practice to benefit the organisation [10]. For example, a receptionist or a salesperson at a hotel may behave unethically and lie to persuade a customer to buy a room or a service in their hotel. This action is often undertaken by an employee to benefit the hotels and increase their sales, especially during crises as in the case of COVID-19 [5]. An employee engaged in this unethical behaviour to avoid mass lay-offs during the pandemic and keep themselves secure in their job [5,6]. Employees may strive to protect themselves and save their job by engaging in this UPoB. Nonetheless, the UPoB is a violation of societal values, standards, and norms [12]. 

This research draws on the social exchange behaviour to examine the direct influence of job insecurity and distributive injustice, which were common in many hotels amid COVID-19, on UPoB among hotel employees. The research also examines the indirect influence on UPoB through turnover intention. More especially, this research examines the mediating role of turnover intention in the relationship between job insecurity, and distributive injustice, which were results of the COVID-19 pandemic on UPoB. The social exchange theory (SET) implies that employees are more likely to exhibit reciprocal attitudes and behaviour similar to those perceived by their peers, supervisors, and managers within their organizations. For example, if employees perceived job insecurity and distributive injustice for their organisation, they could respond by turnover intention and deviant behaviour, including UPoB to avoid lay-off [4]. The unethical pro-organisational behaviour is often explained and viewed by other research through the lens of both SET framework and conservation of resources theory [4,9,10] could assist employees to retain their jobs and at the same time gain acceptance by their organisations and minimize the undesirable perceptions related to this issue. Job security and job retention are all considered resources from the conservation of resources theory [10]. Hence, employees may engage in UPoB to conserve these resources and protect themselves during these uncertain times of the COVID-19 pandemic.

As highlighted above, previous research focused on hotel employees’ attitudes and behaviour amid COVID-19 [2,4,5,6] and investigated employees’ response to job insecurity, distributive injustice, job embeddedness, and turnover intention by engaging in unethical rather than pro-organizational behaviour, which will be undertaken in the current study. This study is among first attempts that examines the UPoB of hotel employees due to the direct effects of job insecurity and distributive injustice and the indirect effect through turnover intention. Previous research studies [5,6] often examines unethical behaviour of employees, not the pro-organisational unethical behaviour, which employees may practice for the sake of the organisation to protect their jobs during the pandemics. The current study contributes the academic body of literature in relation to UPoB, since most literature focuses on unethical behaviour in general rather than pro-organisational behaviour. The research extends literature beyond the major antecedences of UPoB, i.e., job insecurity and distributive injustice, especially because of theCOVID-19 pandemic. The research also highlights the major role of turnover intention in the relationship between job insecurity, distributive injustice, and UPoB. Additionally, the research has some managerial implications for hoteliers to avoid engaging in UPoB, especially during the crisis time, i.e., the COVID-19 pandemic. This study has three research objectives, which are to. First, explore the occurrence of UPoB among hotel employees during the crises, i.e., COVID-19 pandemic, due to job insecurity, distributive injustice, and turnover intention. Second, provide an empirical model examining the influence of job insecurity and distributive injustice on UPoB among hotel employees. Second, examine the role of turnover intention in the relationship between job insecurity, distributive injustice, and UPoB. The study has two main research questions (RQs), which are: decrease the undesirable perceptions related to that prospect.

RQ1: How do both job insecurity and distributive injustice influence the occurrence of UPoB among hotel employees amid COVID-19?

RQ2: What is the intervention role of turnover intention in the relationship between job insecurity, distributive injustice, and UPoB?

The structure of this paper is as follows. Section 2 presents the related literature review, and the research hypothesis and ends with the research conceptual model. Section 3 presents the adopted research methodology. Section 4 presents the data analysis and the results of the study. Section 5 discusses the results and the implications of the study. Section 6 presents the limitations of the study and opportunities for further research. Section 7 shows the research conclusions. 

## 2. Hypothesis Development and Conceptual Framework

### 2.1. Influences of Job Insecurity on Turnover Intention and UPoB

Job insecurity has two main aspects: cognitive aspect, i.e., losing the job, and affective aspect, i.e., negative emotions and concerns related to losing the job [13]. These two aspects were examined in research studies separately or collectively using a combined global measure [14]. Hence, job insecurity refers to both negative emotions and concerns that an employee perceives if his/her continuity in the job is at risk. This perception increases among employees during the crises and pandemic [4], where thousands of hotel employees were subject to lay-off, which is the case of COVID-19 [3]. 

It is not surprising that employees who stayed at their job during the pandemic feel concerned about their job stability. Job insecurity often became a major concern if there were changes in the working environment due to internal or external factors [15,16]. Research [17,18,19] found that the main reason why the hospitality industry has a high turnover rate is because of an unstable working environment. Unsurprisingly, employees who feel insecure in their jobs have an intention to leave for a secure job elsewhere [20]. Recent research studies [4,5,21] have confirmed this assumption that employees who felt less secure in their hotel jobs due to the COVID-19 pandemic have had a higher turnover intention and would like to leave for other jobs. This was also confirmed by earlier research [4,5] that job insecurity of a predictor of turnover intention, especially during downsizing and crisis times. Studies [21,22,23] showed that employees felt insecure in their job due to downsizing or crises, will strive to protect their job even with unethical behaviour, especially if there are limited jobs outside their workplace, such as the uncertainty accompanied by COVID-19 pandemic [1]. 

The SET framework was adopted to explain the relationship between job insecurity and pro-organisational behaviour, such as organisational citizenship behaviour (OCB) as it was found that job insecurity encourages employees to become involved in OCB to avoid losing their jobs [10]. Additionally, the conservation of resources theory was adopted to explain why employees may become engaged in UPoB as it was argued that “employees may engage in pro-organisational behaviour, even if unethical in nature, provided they stand to gain from them” ([10], p. 1185). In other words, employees could respond to job insecurity during the pandemic by engaging in UPoB to benefit their organisation and conserve their resources “their jobs”. Based upon these findings and this discussion, it could be argued that:

**Hypothesis** **1** **(H1).** *Job insecurity has a positive significant influence on UPoB.*

**Hypothesis** **2** **(H2).** *Job insecurity has a positive significant influence on turnover intention.*

### 2.2. Influences of Distributive Justice on Job Insecurity, Turnover Intention, and UPoB

According to the Organisational Justice Theory [24,25], justice has three main components: distributive, intersectional, and procedural. Distributive justice is related to the extent to which outcomes and resources, including compensation and job outcomes, are allocated fairly among employees within the organisation [25]. Hence, distributive justice occurred when an employee compares the received outcomes to his/her his/her peers within the organisation and found an unequal distribution of outcomes, especially if they have similar input. Additionally, Admas [26,27] confirmed that employees often compare their input and output with other employees of the same level of position within the organisation or other organisations. However, if they are different, inequality exists. Research has linked the lack of distributive justice to several psychological impacts on employees, e.g., job stress and mental health [28,29,30].

Limited research studies have examined the indirect influence of justice on the perceptions of job security. For example, the study of Sora et al. [31] showed that the existence of organisational justice makes employees have a lower level of turnover intention and job satisfaction, which implies a lower level of job insecurity. Moreover, distributive injustice was found that leads to unrest and cause stress among employees [29]. All of these issues affect turnover intention [32]. A recent study on hotel workers [32] showed a positive direct influence of distributive justice and disregarding the perceptions of job insecurity. Another recent study [4] found that the existence of distributive injustice in some hotels amid COVID-19 positively affected employees’ turnover intention and their social loafing behaviour. Again, the SET framework strengthens these findings and argument that if employees have perceived distributive justice, they could respond to job insecurity feeling, turnover intention, and UPoB, which also in consistent with the conservation of resources theory [10]. Based on this discussion. It could be hypothesized that:

**Hypothesis** **3** **(H3).** *Distributive injustice has a positive significant influence on job insecurity.*

**Hypothesis** **4** **(H4).** *Distributive injustice has a positive significant influence on turnover intention.*

**Hypothesis** **5** **(H5).** *Distributive injustice has a positive significant influence on UPoB.*

### 2.3. Influences of Turnover Intention on UPoB

Hotels are among the top industries that have a high turnover rate, mainly due to poor and unstable working environments [17,18,19]. Turnover intention refers to the probability of an employee quitting his/her current job. Several predictors were identified for the turnover intention in hotels, including job insecurity [4] organizational justice in general [33], procedural justice [34] and distributive justice [4] in particular, sexual harassment and trust in superior [35], leadership style and organizational commitment [36], job satisfaction [37]. On the other side, turnover intention can r influence job performance [38] and the spread of deviant behaviour as well as social loafing behaviour [4].

As highlighted above UPoB is unethical action, which is often undertaken to benefit the organisation. An employee who has the intention to leave the job because of job insecurity and/or distributive justice is more likely to engage in UPoB to protect their job, particularly if they are aware that they will not have a job outside their organisation. the Conservation of Resources Theory reinforces this assumption that employees can practice UPoB to benefit their organisation and in return receive a benefit from their organisation by saving their resources, i.e., keeping them in their jobs. Based upon these arguments, it could be hypothesized that:

**Hypothesis** **6** **(H6).** *Turnover intention has a positive significant influence on UPoB.*

**Hypothesis** **7** **(H7).** *Turnover intention mediates the relationship between distributive injustice and UPoB.*

**Hypothesis** **8** **(H8).** *Turnover intention mediates the relationship between job insecurity and UPoB.*

**Figure 1:**
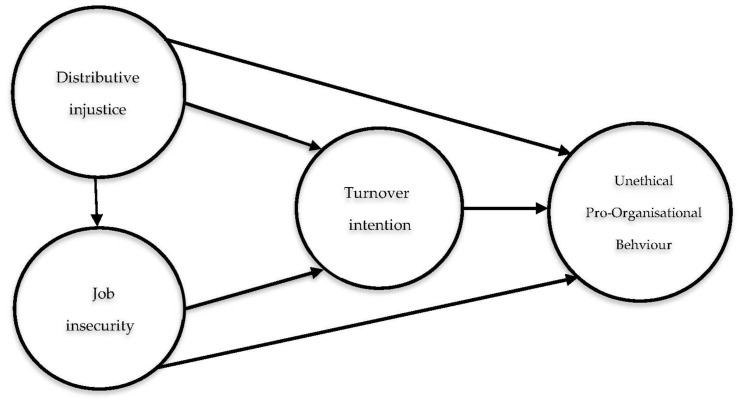
The Research Conceptual Model.

The research framework is summarized in Figure 1.

## 3. Methodology

### 3.1. Research Approach

This study uses a questionnaire survey as its research method. It is a common method for contacting a large sample size of a particular population at a low cost [39]. In their study, Hennessy and Patterson [40] propose that for the survey research method, the authors should first design the research instrument. Consequently, this paper commenced with developing the research instrument.

### 3.2. Questionnaire Designing

The questionnaire was designed in five main sections. Section one targeted the respondent characteristics such as name, age, education, gender, and working experience. Section two asks about the unethical pro-organizational behaviour. Section three contains the distributive injustice questions, while section four reflected the employee’s turnover intention, and finally section five contains the job insecurity questions. 

In this case, 11 academics and 13 employees were asked to complete the questionnaire in order to ensure its clarity and reliability during the pilot phase. The questionnaire’s content was not changed. The questionnaire declares the collected data to be anonymous and confidential. Since the questionnaire is self-reporting, common method variance (CMV) may be an issue [41]. To deal with CMV, Harman’s single-factor analysis was used, with the extracted factors constrained to 1.00 in an exploratory factor analysis (EFA) test using SPSS (IBM, Armonk, NY, USA) with no rotation. As only one factor explained 32% (less than 50%) of the variance, CMV is not an issue [41].

### 3.3. Construct Measures 

The study measures were developed following an extensive survey and review of previously published theoretical measures. This survey generates four dimensions, each with its related set of variables, which were tailored to suit the hospitality industry. The measures were developed using a five-point Likert-type scale, with 1 representing “strongly disagree” and 5 representing “strongly agree”. Job insecurity (JobInsc, *a* = 0.906) was operationalized by six variables three of them measure the quantitative aspects of job insecurity while the other three variables measure the qualitative aspects of job insecurity, the items were established by Hellgren et al. [42] and employed by Elshaer and Azazz [5], example item incorporates “I worry about being able to keep my job”. Furthermore, Colquitt (2001) four variables scale of distributive justice (D_Injustice, *a* = 0.908) was modified to operationalize distributive injustice, example item “I feel that the outcome process is inappropriate for the work I completed”. Additionally, UPOB (*a* = 0.919) was measured by seven- items obtained from Umphress et al. [9] and employed by Elshaer and Azazz [5], example item “If it would benefit my organization, I would withhold negative information about my company or its products from customers and clients”. Finally, turnover intention (Trn_Inten, *a* = 0.914) scale was derived from Singh et al. [43]; Karatepe [44]; Elshaer and Saad [23] and operationalized with three reflective items to indicate the employees’ desire to change career and switch to a new field. 

### 3.4. Data Collection

A simple random sample of 700 employees working in hotels in Saudi Arabia’s Eastern Province were selected to complete the designed questionnaire. The Eastern Province of Saudi Arabia is the largest in the Kingdom and is extremely well-known for its long, beautiful coasts that are located in the Persian Gulf. According to Saudi Vision 2030, the hotel industry will be one of the primary contributors to the diversification of the Saudi economy apart from oil. Amid the COVID-19 pandemic, employees in the hotel industry suffer from the feeling of job insecurity and distributive injustice due to the lockdown and layoff decisions in this sector. Consequently, employees in the hotel industry in Saudi Arabia are a good and adequate context that serves the purpose of the current study to test the effect of distributive injustice on UPoB through the mediating role of job insecurity and turnover intention. 

The questionnaire was circulated to the targeted sample during November and December 2021. The research team uses its vast personal networks to drop and collect data, as this method yielded the highest response rate [45]. The research team was able to distribute 700 questionnaires, from which 660 responses were restarted, while 10 surveys were excluded because of the missing answers, generating a valid 650 surveys for further analysis, with a 92.5% response rate. The missing data in our study is less than 2% in a random pattern and were handled through the imputation method suggested by Tabachnick and Fidell [46] who argued that if the missing data did not exceed 5%, the problems arise from missing data are not serious and nearly any method for handling it will produce similar findings [46].

The sample size of 650 is adequate and considered more than enough for SEM analysis as it fulfils Nunnally’s [47] suggestion of a minimum of 10 surveys per the measure’s items (the measurement in this study has 20 items, hence exceeding the recommended sample size of 200); and it matches Hair et al.’s [48] criteria of at least 100 to 150 respondents to generate an adequate solution of MLE “maximum likelihood estimation”. Additionally, according to the Krejcie and Morgan [49] suggestions, if the study population surpasses 1,000,000, the lowest needed sample size is 384, in this study the sample size of 650, surpasses the suggestions. An Independent sample t-test procedure was conducted to compare the early, and late replies mean. Non-response bias was not a problem, no significant differences *p* > 0.05) were detected giving evidence that non-response bias was not an issue [47].

## 4. Data Analysis

### 4.1. Descriptive Statistics

As shown in Table 1. the vast majority (61.5%) of the participants were male and married (69%). More than half of the respondents (55%) were between the age of 30 to 45 years. Approximately 65% of those surveyed were former college students. 444 respondents (68%) had working experience for less than five years, while 206 (32%) served between 6 and 15 years.

Table 2 presents the descriptive properties of the study respondents as well. The mean (M) values of the respondents ranged between 3.33 and 3.87, and the standard deviation (S.D.) values ranged between 0.187 and 1.239, giving evidence that the data was more spread out and less concentrated around the mean value [47]. Additionally, the skewness and kurtosis scores of the data distribution are included in Table 2, with no values exceeding the score of −2 or +2, indicating that the data has a normal distribution [48].

### 4.2. Confirmatory Factor Analysis (CFA)

To assess the validity and reliability of the employed scale, all independent and dependent factors, as well as their associated reflective variables, were subjected to first-order CFA with AMOS graphics with maximum likelihood estimation (MLE) procedures. As recommended by Haire et al. [48]; Bryman and Cramer [50]; Kline [51]; Anderson, and Gerbing [52]; and Fornell and Larcker [53], different goodness of fit (GoF) criteria were used to evaluate the model’s fit to the data., containing chi-square divided into the degree of freedom “normed chi-square”, “root means square error approximation” (RMSEA), “Comparative Fit Index” (CFI), and “Tucker Lewis index” (TLI). The Amos GoF output confirmed that the CFA exhibited adequate and satisfactory fit to data (see Table 3). The scale reliability was evaluated with Cronbach’s alpha values (showed in the measurement section) and “composite reliability” (CR). Table 3 displays the CR values for the four study dimensions: job insecurity (0.957), turnover intention (0.903), distributive injustice (0.969), and UPOB (0.979), all CR values exceeded the threshold criteria of 0.7 as suggested by Fornell and Larcker [53] indicating that the data has a satisfactory internally consistent.

Furthermore, the employed scale convergent validity was satisfactory and adequate for two key reasons: (1), all the standardized factor loadings (SFL) were acceptable and adequate with a high significant *p*-value of less than 0.001, as depicted in Table 3. Table 3 indicates that all SFL scores ranged between 0.82 and 0.97, surpassing the threshold value of 0.50 [48]. (2), the AVE (average variance extracted) values for all employed four dimensions: Job insecurity (0.790), turnover intention (0.756), distributive injustice (0.887), and UPOB (0.869), surpassed the value of 0.50, showing adequate and acceptable convergent validity [48] (see Table 3).

Further to that, the discriminant validity was acceptable due to two key conditions as suggested by Hair et al. [48]; Bryman and Cramer [50]; Anderson, and Gerbing [52]: (1) The MSV “maximum shared variance” values should not exceed the AVE values, as exposed in Table 3; (2) The AVE square root values for the four employed dimension (the bold diagonal values) surpassed the values of dimensions intercorrelation (values below the bold diagonal values) as depicted in Table 3. 

### 4.3. Structural Equation Modeling (SEM)

**Figure 2:**
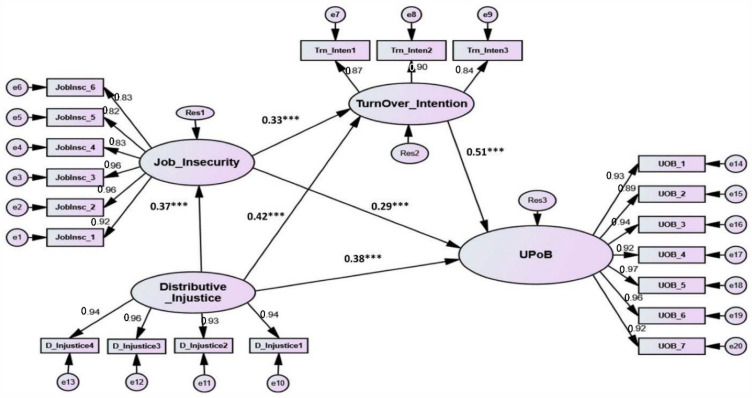
The structural Model. Note: *** *p* < 0.001.

In this study, the researchers used a confirmatory two-step strategy, which involved conducting an extensive literature review in order to develop a theoretical conceptual model, and then collecting observed data in order to determine whether or not it corresponded to the previously specified theoretical conceptual model [52]. The theoretical proposed structural model is either rejected or approved in this strategy based on its ability to satisfy a model fit condition. The structural proposed model fit the observed data well, based on SEM output: χ^2^ (164, N = 650) =783.756, *p* < 0.001, normed χ^2^ = 4.779, RMSEA= 0.039, SRMR = 0.0370, CFI = 0.916, TLI = 0.926, NFI = 0.917 (as indicated in Table 4). The study hypotheses were evaluated after obtaining an adequate model fit to the data. The proposed hypotheses are depicted in Figure 2, with each path representing a distinct hypothesis.

This study suggested eight main hypotheses. The results demonstrate that job insecurity positively and significantly associated with UPOB (β = 0.29. t-value = 4.323, *p* < 0.001); turnover intention (β = 0.33, t-value = 7.175, *p* < 0.001); and distributive injustice (β = 0.37, t-value = 8.443, *p* < 0.001) hence, Hypotheses H1, H2, and H3 were supported. Similarly, the SEM results displays that distributive injustice is positively and significantly associated with turnover intention (β = 0.42, t-value = 9.789, *p* < 0.001) and UPOB (β = 0.38, t-value = 8.971, *p* < 0.001). Finally, turnover intention was found to has significant and positive impacts on UPOB (β = 0.51, t-value = 12.154, *p* < 0.001) supporting Hypothesis H6.

Additionally, the results explored the mediation effects of turnover intention and distributive injustice in the relationships between job insecurity and UPOB. All path coefficients (direct and indirect) in the tested model were found to be positive and significant therefore complementary mediation is confirmed as suggested by Zhao et al. [54], thus Hypotheses H7 and H8 were supported. Furthermore, the SEM results demonstrate more evidence that supports the mediation effects of turnover intention and distributive injustice in the relationship between job insecurity and UPOB, as the direct positive significant impacts of job insecurity on UPOB was increased from (β = 0.29, *p* = 0.001) to a total effect of 0.54 with significant *p* > 0.001 [48]. Table 4 also demonstrates that the explanatory power (R2) of all paths (R2 = 0.50) explains 50% of the variance in UPOB.

The preceding results were confirmed by computing the specific indirect estimation using a bootstrapping method from Amos estimates to verify the mediation effects of job insecurity and turnover intention in the relationships between distributive injustice and UPOB. Four specific indirect estimates have emerged as depicted in Table 5. The specific indirect path from distributive injustice to UPOB through job insecurity showed a lower (0.286) and an upper score (0.383) that generated a significant (*p* > 001) standardized indirect regression of 0.304. Likewise, the indirect path from distributive injustice to UPOB through turnover intention demonstrated a lower (0.301) and an upper score (0.472) that produced a significant (*p* > 001) indirect regression of 0.407. Additionally, the indirect path from job insecurity to UPOB through turnover intention showed a lower (0.322) and an upper score (0.480) that generated a significant (*p* > 390) standardized indirect regression of 0.304. Finally, the indirect path from distributive injustice to UPOB through job insecurity and turnover intention possesses a lower (0.342) and an upper value (0.501) that created a significant (*p* > 001) standardized estimate of 0.410. Thus, further supporting Hypotheses H7, and H8. 

## 5. Discussion and Implications

The issue of unethical behaviour has drawn the attention of many researchers [5,6,7]. In the same line, UPoB has attracted the attention the many researchers [8,9,10]. Nonetheless, research amid COVID-19 pandemic often focused on unethical behaviour rather than UPoB, which is crucial for organisations and their success. This research is an attempt to examine the direct influence of both job insecurity and distributive injustice on UPoB among hotel employees and the indirect influence through turnover intention. The results supported all the research hypotheses in the conceptual model (Figure 1). First, the results demonstrate that both distributive injustice and job insecurity positively, significantly, and directly influence UPoB. This finding supports recent study on hotel employees that both job insecurity and distributive injustice significantly influence social loafing behaviour among employees, which is unethical behaviour [4]. Moreover, this result is in line with the work of Ghosh [10], who also found that job insecurity has a positive significant influence on the pro-organisational behaviour of employees to protect themselves and their jobs. This finding also is in coincidence with the SET framework [55,56] and Conservation of Resources Theory [57] and previous research pre COVID-19 [10] that employees responded to their higher perceptions of job insecurity in general, but in this research because of COVID-19 pandemic, by engaging in UPoB to deal with this stress and problem and benefit their organisation, which in return help them protect themselves and keep their resources, i.e., jobs. The results of current research confirm that employees practice this antisocial behaviour “UPoB”, which they really understand that it is bad for good results from their point of view, which is to benefit their organisations. However, they really want to benefit themselves by keeping their jobs. Despite this unethical behaviour, however, could benefit the organisation in the short term, it will have a negative influence on the organisation in the long term [7,9]. Additionally, the occurrence of this behaviour will defiantly affect the behaviour of other employees [34], which could spread this UPoB among all employees. 

The results also confirmed that job insecurity positively and significantly influences turnover intention. These results have supported previous research [20] that employees who perceive job insecurity will have an intention to turnover and leave for a secure job elsewhere. The results are also in line with other recent research studies [4,5] that employees who felt less secure in their hotel jobs due to COVID-19 pandemic have had a higher turnover intention and want to leave for other jobs. Moreover, the results showed that distributive injustice positively and significantly influences job insecurity. The literature [28,29,30] confirmed that the lack of distributive justice has several psychological impacts on employees, e.g., job stress and mental health. Nonetheless, the current research approved direct, positive, and significant influence of distributive injustice on job security among hotel employees. This means that when employees perceived unequal distribution of resources during the COVID-19 pandemic, they have had a higher perception of job insecurity, which could also lead to other negative consequences, e.g., turnover intention and UPoB. Furthermore, the results showed that distributive injustice has a significant positive influence on hotel employees’ turnover intention. These results are in agreement with the work of Alyahya et al. [4], who also found a positive direct influence of distributive injustice amid COVID-19 on turnover intention among hotel employees.

The results confirmed a direct, positive, and significant influence on turnover intention, which was the results of COVID-19 on UPoB. This is a very interesting finding because employees engaged in pro-organisation unethical behaviour because they were forced to leave their jobs. The last option for them to save their jobs during this pandemic with mass-lay off is to engage in this UPoB to protect themselves and save their resources (in our case is their jobs). Furthermore, turnover was found to have a mediating role in the relationship between distributive injustice, job insecurity, and UPoB. The research confirms that an employee who has the intention to leave the job because of job insecurity and/or distributive injustice are more likely to engage in UPoB to protect their job, particularly if they are aware that they will not have a job outside their organisation, but they are directed towards turnover intention. 

The research has numerous theoretical implications for scholars and managerial implications for hoteliers. The research contributed to the literature in relation to the pro-organisational behaviour, especially UPoB. The research showed that to deal with distributive injustice and job insecurity because of the pandemic, hotel employees engaged in UPoB to benefit their organisation and protect their jobs at this uncertain time of COVID-19. Literature (e.g., [7,8,9]) showed that employees could be engaged in unethical behaviour if they felt insecure; however, the literature has limited studies [10] to confirm that employees could be engaged in pro-organisational behaviour, especially UPoB to defend themselves and their resources (in our case their jobs) because of job insecurity and distributive injustice amid COVID-19 pandemic. Additionally, the turnover intention due to the COVID-19 also supports this unethical pro-organizational behaviour. More specifically, turnover intention was found to have a mediating effect on the relationship between distributive injustice, job insecurity, and pro-organisational behaviour, especially the unethical one or UPoB. The current research provided an empirical structural model, which confirmed the direct influence of job insecurity and distributive injustice on UPoB among hotel employees and the indirect influence through turnover intention.

Senior managers in hotels need to recognize that their employees are the most important resource that they have; hence investing in them and retaining them has to be their main priority. Manager should also recognize that their employees would be respond to the threat of their job insecurity and distributive justice by engaging not just unethical behaviour but in pro-organisational one. Employees would argue that they undertake this behaviour for the sake of the organisation, however, they ultimately want to save their job during this uncertain time. Additionally, managers should also understand that despite the occurrence and spread of this UPoB that could benefit the organisation in the short term, it will have many negative consequences in the long term. These consequences will negatively affect employees’ behaviour and organisation overall. Therefore, hotel managers should spend all endeavours to equally distribute the resources and outcomes among their workers and ensure they are satisfied with this. Senior hotel managers need to properly manage the threats that lead to job insecurity and turnover intention among their employees to avoid their involvement in any unethical behaviour, especially the UPoB.

## 6. Conclusions

The current study confirmed all the hypothesised relationships. A positive and direct influence of both distributive injustice and job insecurity on turnover intention and UPoB among hotel employees amid COVID-19 pandemic. Turnover also has a direct influence on UPoB and a mediating role in distributive injustice, job insecurity, and UPoB. The conclusions of the current research study are as follow. First, the positive and direct influence of both distributive injustice and job insecurity on turnover intention amid the COVID-19 pandemic reflects that employees who perceived job insecurity and distributive injustice have a higher intention to leave the job and look for another secure job. Second, the positive and direct influence of distributive injustice and job insecurity on UPoB confirms that if employees responded to their feeling of distributive injustice and job insecurity, because of COVID-19 pandemic, by engaging in UPoB to deal with this stress and problem and benefit their organisation, which in return help them protect themselves and keep their jobs. However, this behaviour could have negative consequences on the organisation in the long term. Third, interestingly, the results showed a positive direct influence of distributive injustice on job insecurity, which confirms that when employees felt inadequate distribution of resources during the COVID-19 pandemic, they responded with higher perceptions of job insecurity. Fourth, the results confirmed a direct, positive, and significant influence of turnover intention, which was the results of COVID-19, on UPoB. This means that employees engaged in pro-organisation unethical behaviour because they were forced to leave their jobs. They want to protect themselves and save their job since there were limited or no opportunities outside the organisation. Therefore, they responded by engaging in UPoB. Finally, turnover intention has a mediating effect. Turnover intention was found to increase the influence of distributive injustice and job insecurity on UPoB. Thus, the proper management of these factors, i.e., distributive injustice, job insecurity, and turnover intention, will help organisation control the occurrence and spread of UPoB among their employees. 

## 7. Limitations and Future Research Prospects

Despite the value of the current research, it has a number of shortcomings similar to many other studies. All of these limitations present potential topics for upcoming research studies. First, the study was conducted in the Eastern Province of the kingdom of Saudi Arabia. Hence, the results may affect the Saudi culture and/or the organisational culture in these hotels, which was not examined in the current study. However, this gives the opportunities for researchers to examine this issue further. Second, the findings revealed that turnover intention fairly mediated the effect of job insecurity, distributive injustice, and unethical pro-organizational behaviour. Future research studies can examine more mediating variables (e.g., financial pressure, trust in supervisors [58], and job embeddedness) that can affect the relationship between distributive injustice, job insecurity, and unethical pro-organizational behaviour. Third, future research can also address decision-makers practices to reduce UPoB and suggest methods to control it. Fourth, because the data collected were cross-sectional, the causal relationship between latent variables could not be fully established. Future studies should collect longitudinal objective data or use a different data source to validate the study model. Finally, future studies can use a multi-group analysis approach to validate and compare the current study’s findings with data collected from different contexts (industry/country).

## Figures and Tables

**Table 1:** Respondents’ Demographics.

		N = 650	Percentage (%)
Gender	Female	251	38.5%
	Male	399	61.5%
Marital status	Married	449	69%
	Unamarried	201	31%
Age	<21 years old	33	5%
	22 to 29	65	10%
	31–45 years old	357	55%
	46 to 60 years old	162	25%
	>61 years old	33	5%
Working experience	<5 years of experience	444	68.3%
	6–10 years of experience	141	21.7
	11- 15 years of experience	65	10

**Table 2:** Descriptive data (Mean, Standard Deviation, Skewness and Kurtoses values).

Abbreviation	Items	M	S.D.	Skewness	Kurtosis
**Job Insecurity [41] (*a* = 0.906)**					
JobInsc_1	“I am worried that I will have to leave my job before I would like to”.	3.24	1.041	−0.384	−0.321
JobInsc_2	“I worry about being able to keep my job”.	3.24	1.05	−0.346	−0.392
JobInsc_3	“I am afraid I may lose my job shortly”.	3.27	1.01	−0.378	−0.273
JobInsc_4	“I worry about getting less stimulating work tasks in the future”.	3.22	1.08	−0.448	−0.281
JobInsc_5	“I worry about my future wage development”.	3.23	1.07	−0.454	−0.259
JobInsc_6	“I feel worried about my career development in the organization”.	3.22	1.09	−0.472	−0.255
**Turn over Intention [23,42,43] (*a* = 0.914)**					
Trn_Inten1	“I often think about leaving that career”.	3.65	1.185	−0.457	−0.868
Trn_Inten2	“It would not take much to make me leave this career”.	3.62	1.134	−0.391	−0.848
Trn_Inten3	“I will probably be looking for another career soon”.	3.64	1.148	−0.399	−0.882
**Distributive injustice [25] (*a* = 0.908)**					
D_Injustice1	“I feel that the outcome process does not reflect the effort I have put into my work.”	3.56	1.243	−0.371	−0.976
D_Injustice2	“I feel that the outcome process is inappropriate for the work I completed.”	3.55	1.239	−0.363	−0.971
D_Injustice3	“I feel that the outcome process does not reflect what I have contributed to the organization.”	3.55	1.239	−0.359	−0.973
D_Injustice4	“I feel that the outcome process is unjustified, given my performance.”	3.52	1.284	−0.380	−1.008
**UPOB [9] (*a* = 0.919)**					
UPOB_1	“If it would help my organization, I would misrepresent the truth to make my organization look good.”	3.87	1.187	−1.100	0.369
UPOB_2	“If it would help my organization, I would exaggerate the truth about my “company’s products or services to customers and clients.”	3.76	1.231	−0.980	−0.020
UPOB_3	“If it would benefit my organization, I would withhold negative information about my company or its products from customers and clients.”	3.80	1.205	−1.021	0.181
UPOB_4	“If my organization needed me to, I would give a good recommendation on the behalf of an incompetent employee in the hope that the person will become another organization’s problem instead of my own.”	3.79	1.225	−1.040	0.148
UPOB_5	“If my organization needed me to, I would withhold issuing a refund to a customer or client accidentally overcharged.”	3.76	1.223	−0.969	0.033
UPOB_6	“If needed, I would conceal information from the pUPoBlic that could be damaging to my organization.”	3.74	1.252	−0.987	−0.014
UPOB_7	“I would do whatever it takes to help my organization.”	3.77	1.221	−0.980	0.031

**Table 3:** First order factor analysis Convergent and discriminant validity.

Factors and Items	Loading	CR	AVE	MSV	1	2	3	4
**1-Job Insecurity**		0.957	0.790	0.016	**0.889**			
JobInsc_1	0.924							
JobInsc_2	0.961							
JobInsc_3	0.960							
JobInsc_4	0.826							
JobInsc_5	0.818							
JobInsc_6	0.829							
**2-Turnover Intention**		0.903	0.756	0.012	0.109	**0.925**		
Trn_Inten1	0.868							
Trn_Inten2	0.903							
Trn_Inten3	0.836							
**3-Distributive injustice**		0.969	0.887	0.025	0.035	0.018	**0.948**	
D_Injustice1	0.940							
D_Injustice2	0.935							
D_Injustice3	0.957							
D_Injustice4	0.936							
**4-Unethical pro-organizational behavior**		0.979	0.869	0.025	0.125	0.027	0.158	**0.929**
UPOB_1	0.933							
UPOB_2	0.888							
UPOB_3	0.940							
UPOB_4	0.917							
UPOB_5	0.966							
UPOB_6	0.958							
UPOB_7	0.921							

Note: CR: composite reliability; AVE: average variance extracted; MSV: maximum shared value; Bold diagonal values: the square root of AVE for each dimension; below diagonal values: intercorrelation between dimensions. Model GoF: “(χ^2^ (164, N = 650) = 591.07, *p* < 0.001, normed χ^2^ = 4.021, RMSEA = 0.022, SRMR = 0.0321, CFI = 0.916, TLI = 0.986, NFI = 0.912, PCFI = 0.701 and PNFI = 0.698)”.

**Table 4:** The structural model’s results.

Hypotheses				Beta (β)	C-R (T-Value)	R^2^	Results of Hypotheses
H1	Job Insecurity	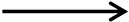	UPoB	0.29 ***	4.323		Supported
H2	Job Insecurity	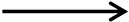	Turnover intention	0.33 ***	7.175		Supported
H3	Distributive injustice	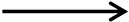	Job Insecurity	0.37 ***	8.443		Supported
H4	Distributive injustice	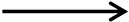	Turnover intention	0.42 ***	9.789		Supported
H5	Distributive injustice	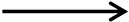	UPoB	0.38 ***	8.971		Supported
H6	Turnover intention	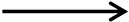	UPoB	0.51 ***	12.154		Supported
H7	Job Insecurity 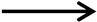 Turnover intention 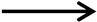 UPoB			Path 1: β =0.33 *** t-value = 7.175 Path 2: β = 0.51 *** t-value = 12.154			Supported
H8	Distributive injustice 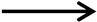 Job Insecurity 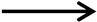 UPoB			Path 1: β =0.37 *** t-value =8.443 Path 2: β = 0.29 *** t-value = 4.323			Supported
	Turnover intention					0.30	
UPoB						0.50	

Model GoF: χ^2^ (164, N = 650) =783.756, *p* < 0.001, normed χ^2^ = 4.779, RMSEA = 0.039, SRMR = 0.0370, CFI = 0.916, TLI = 0.926, NFI = 0.917, PCFI = 0.707 and PNFI = 0.701. *** *p* < 0.001.

**Table 5:** Specific indirect estimate.

Specific Indirect Paths	Unstandardized Estimate	Lower	Upper	*p*-Value	Standardized Estimate
Distributive injsutice → job insecurity → UPoB	0.325	0.286	0.383	0.001	0.304 ***
Distributive injsutice → Turnover intention → UPoB	0.431	0.301	0.472	0.001	0.407 ***
job insecurity → Turnover intention → UPoB	0.401	0.322	0.480	0.001	0.390 ***
Distributive injsutice → job insecurity → Turnover intention → UPoB	0.421	0.342	0.501	0.001	0.410 ***

*** *p* < 0.001.

## Data Availability

Data is available upon request from researchers who meet the eligibility criteria. Kindly contact the first author privately through the e-mail.
